# Thermodynamics of the DNA Damage Repair Steps of Human 8-Oxoguanine DNA Glycosylase

**DOI:** 10.1371/journal.pone.0098495

**Published:** 2014-06-09

**Authors:** Nikita A. Kuznetsov, Alexandra A. Kuznetsova, Yuri N. Vorobjev, Lev N. Krasnoperov, Olga S. Fedorova

**Affiliations:** 1 Siberian Branch of the Russian Academy of Sciences, Institute of Chemical Biology and Fundamental Medicine, Novosibirsk, Russia and Department of Natural Sciences, Novosibirsk State University, Novosibirsk, Russia; 2 Department of Chemistry and Environmental Science, New Jersey Institute of Technology, Newark, New Jersey, United States of America; Florida International University, United States of America

## Abstract

Human 8-oxoguanine DNA glycosylase (hOGG1) is a key enzyme responsible for initiating the base excision repair of 7,8-dihydro-8-oxoguanosine (oxoG). In this study a thermodynamic analysis of the interaction of hOGG1 with specific and non-specific DNA-substrates is performed based on stopped-flow kinetic data. The standard Gibbs energies, enthalpies and entropies of specific stages of the repair process were determined via kinetic measurements over a temperature range using the van’t Hoff approach. The three steps which are accompanied with changes in the DNA conformations were detected via 2-aminopurine fluorescence in the process of binding and recognition of damaged oxoG base by hOGG1. The thermodynamic analysis has demonstrated that the initial step of the DNA substrates binding is mainly governed by energy due to favorable interactions in the process of formation of the recognition contacts, which results in negative enthalpy change, as well as due to partial desolvation of the surface between the DNA and enzyme, which results in positive entropy change. Discrimination of non-specific G base versus specific oxoG base is occurring in the second step of the oxoG-substrate binding. This step requires energy consumption which is compensated by the positive entropy contribution. The third binding step is the final adjustment of the enzyme/substrate complex to achieve the catalytically competent state which is characterized by large endothermicity compensated by a significant increase of entropy originated from the dehydration of the DNA grooves.

## Introduction

**Figure 1:**
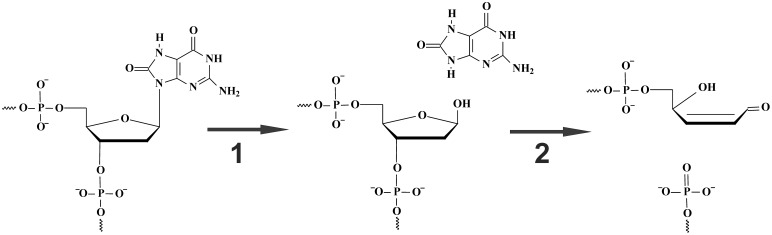
The chemical steps of hOGG1 catalysis. Step 1: oxoG-base removal and formation of abasic product, step 2: β-elimination of 3′-phosphate resulting in formation of the nicked product.

DNA is continuously damaged by reactive oxygen species (ROS) generated by UV light, ionizing radiation and during metabolism [1]–[5]. Among the various products of oxidative stress, 7,8-dihydro-8-oxoguanosine (oxoG) is the most commonly found. It is a pre-mutagenic DNA lesion since oxoG is able to mispair with adenine, thus generating G/C to T/A transversion mutations [6]. As is the case of most oxidized bases, oxoG is primarily removed in the base excision repair (BER) pathway. This pathway is initiated by the recognition of the modified bases by specific DNA glycosylases. In human cells, 8-oxoguanine DNA glycosylase hOGG1 accomplishes the excision of oxoG residues. HOGG1 belongs to the HhH-GPD superfamily of DNA glycosylases containing conserved structural helix-hairpin-helix and GPD motifs [7]–[10]. HOGG1 is a bifunctional enzyme, possessing DNA glycosylase activity (hydrolysis of the N-glycosidic bond of the damaged nucleotide resulting in formation of the abasic product, Fig. 1) and AP lyase activity (elimination of the 3′-phosphate, often referred to as β-elimination resulting in formation of the nicked product, Fig. 1). In contrast to several other bifunctional DNA glycosylases, the AP lyase activity of hOGG1 is much weaker than its glycosylase activity [11]–[14].

**Figure 2:**
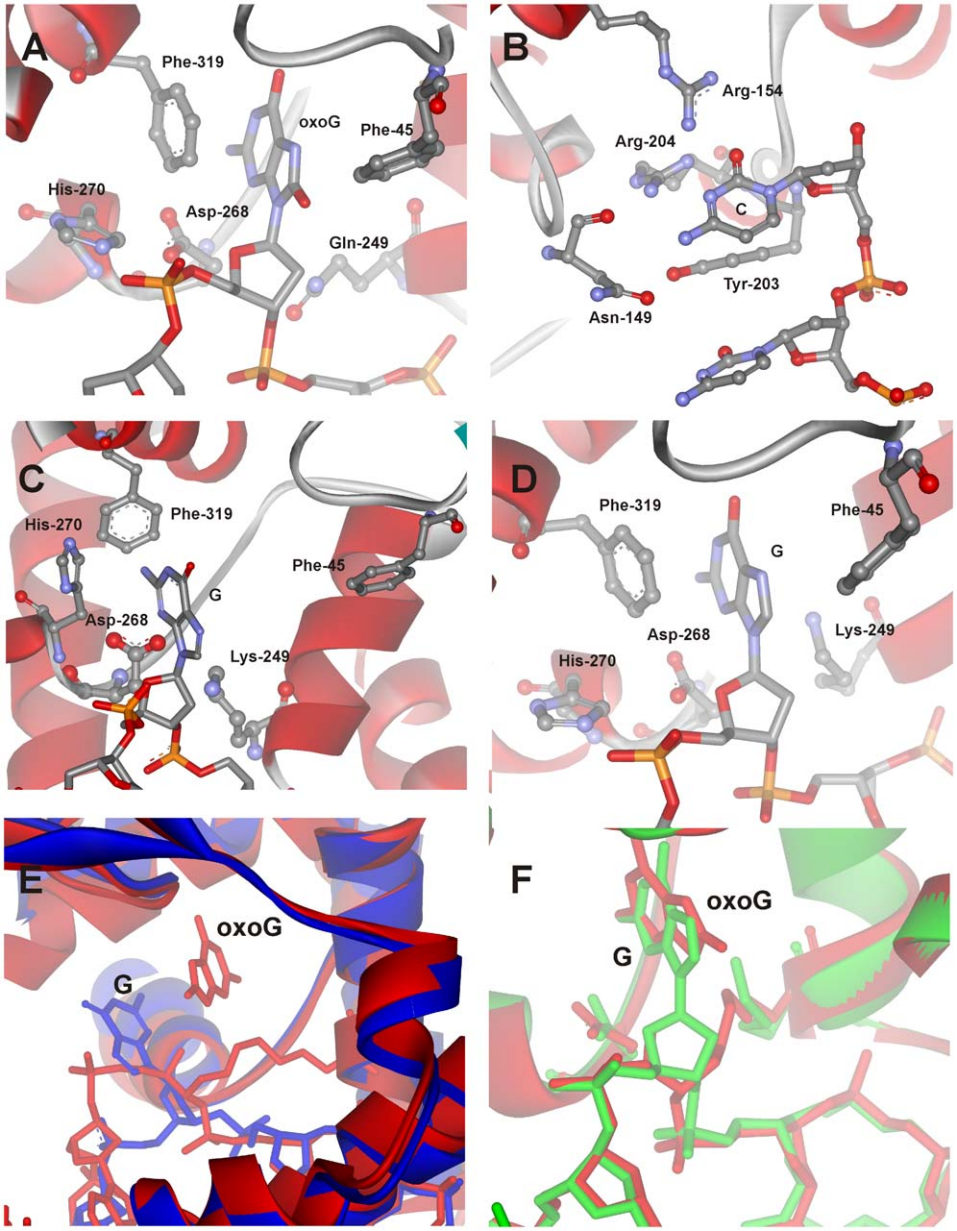
Close-up view of the active site of the hOGG1 complexes with damaged and undamaged DNA. (A) The lesion recognition complex of hOGG1 bound to DNA with the oxoG base inserted in the active site (PDB ID: 1YQR, [17]), (B) specific contacts of enzyme with the cytosine which is located opposite to oxoG base (PDB ID: 1EBM, [9]), (C) the complex of hOGG1 bound to the DNA with the G base inserted in the exo-site (PDB ID: 1YQK, [17]), (D) the complex of hOGG1 bound to the DNA, with the G base inserted in the active site (PDB ID: 3IH7, [19]). The overlays of the trapped catalytic complex hOGG1/oxoG-substrate (red, 1HU0, [16]) with either (E) the complex of hOGG1 bound to DNA with the G base inserted in the exo-site (blue, 1YQK, [17]) or (F) the complex of hOGG1 bound to DNA, with the G base inserted in the active site (green, 3IH7, [19]).

The 3D structures of free hOGG1 and its covalent complexes with DNA have been determined by X-ray crystallography [9], [10], [15]–[19]. The structural data indicate that the conformations of both enzyme and DNA undergo significant changes upon binding. The enzyme forms non-specific electrostatic and hydrophobic contacts with the sugar-phosphate backbone. In the specific lesion recognition complex the DNA is kinked by 70°. The oxoG base is fully flipped out from the DNA helix and deeply inserted into the active site of the enzyme (Fig. 2A). Amino acids Arg-154 and Arg-204 inserted in the DNA helix participate in bidentate hydrogen-bonding interactions with the cytosine base, located opposite to the damaged base (Fig. 2B). Additionally, amino acids Asn-149 and Tyr-203 are inserted into the abasic void released due to flipping out of the oxoG residue.

According to the structure of the complex of hOGG1 with a non-specific DNA, the non-damaged G base is held in the semi-everted transition state (Fig. 2C) [17]. In this state the base is located in the exo-site at the distance of ca. 5 Å from the catalytic enzyme pocket. Nevertheless, in a recent paper from Verdine’s laboratory, a different structure of the complex hOGG1 with non-damaged DNA is proposed (Fig. 2D) [19]. It was shown that although a non-damaged G base can be placed into the active site of hOGG1 approximately in the same position as an oxoG base in the lesion recognition complex (Fig. 2E and 2F), the catalytic step does not occur with DNAs containing non-damaged G base [16]. Therefore the question arises - what is the mechanism of recognition of the damaged nucleotide?

**Figure 3:**
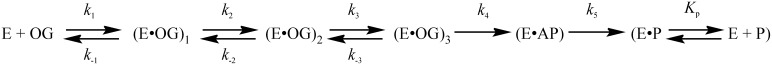
Kinetic mechanism of hOGG1 processing of the oxoG-substrate. Here E is hOGG1; OG is the oxoG-substrate; (E•OG)_n_ are different enzyme-substrate recognition complexes; E•AP is the complex of E with the abasic site formed in the course of N-glycosylase reaction; E•P is the enzyme-product complex formed in the AP-lyase reaction; P is the final free reaction product; *k*_i_ and *k*_–i_ are the rate constants of the forward and backward individual processes.

To address this issue, in the previous studies we investigated the conformational dynamics of hOGG1 and DNA substrates during the catalytic cycles using fluorescence detection [14], [20]. The enzyme molecule contains 10 fluorescent tryptophan amino acids, located in several parts of the spatial structure. The fluorescence changes of Trp residues in hOGG1 as well as 2-aminopurine (aPu) residues in the DNA substrates have been detected during catalytic cycle at single-turnover conditions. It was shown that the interaction proceeds through multiple conformational transitions, attributed to sequential lesion recognition process, catalytic steps and release of the product from the DNA complex with enzyme [14], [20]. A kinetic scheme (Fig. 3) containing three initial reversible recognition steps, two irreversible chemical steps and final reversible step was proposed to explain the observations. The structural nature of these steps was suggested based on the analysis of several mutant forms of hOGG1 as discussed in detail in [21]. These studies resulted in atomic-level structural and kinetic information to understand the sequence of hOGG1-DNA interactions during the recognition of oxoG base, and provided insights into the roles of individual amino acids in this process. The analysis of the fluorescence kinetic data lead to the conclusion that the first step of the oxoG-DNA repair is a non-specific binding process which consists of C base pulling by Arg-154/Arg-204, pushing of Tyr-203 into DNA helix, electrostatic interaction between Lys-249 and the damaged nucleotide, oxoG flipping out in the exo-site, and the interaction of His-270 with the everted base [21]. During the second step the oxoG base is everted into the active site and is held there due to the π–π stacking with the aromatic ring of Phe-319. After that, His-270 interacts with phosphate group of the everted nucleotide and forms hydrogen bonds between Arg-154, Arg-204, Asn-149 and the C base. The third DNA damage recognition step is most likely associated with the full insertion of Tyr-203 in the duplex and oxoG interaction with Gly-42.

Understanding of the mechanism of the DNA damaged base recognition by hOGG1 may be improved by elucidation of the thermodynamics of the binding and cleavage steps via determination of their Gibbs free energy (Δ*G*°), enthalpy (Δ*H*°) and entropy (Δ*S*°). Correlation of the thermodynamic data with the proposed sequential mechanism of oxoG-substrate binding and cleavage might result in the quantitative description of the driving forces that govern the structural adjustment of the enzyme and DNA. In the present work, the thermodynamic parameters of the specific steps occurring in the time range 1 ms–1000 s during oxoG base recognition and removal by hOGG1 were obtained via the temperature-dependent fluorescence kinetic data using the van’t Hoff and Eyring equations [22]. The rate constants of the forward and reverse reactions as well as the resulting equilibrium constants of the individual steps in a multi-step reaction mechanism were determined based on the temporal 2-aminopurine (aPu) fluorescence traces recorded at different temperatures. This fluorescent analog of purine bases is sensitive to the DNA conformation and was widely used for the studies of structural changes in DNA (see [23], [24] for details).

## Materials and Methods

### Protein Expression and Purification

To purify hOGG1 expressed as a recombinant protein, 1 liter of *Escherichia coli* strain Rosetta II(DE3) (Invitrogen, France) carrying the pET28c(+) hOGG1 construct was grown in LB broth with 25 µg/ml kanamycin at 25°C until A600 = 0.6–0.7 and induced overnight with 0.2 mM isopropyl-β-D-thiogalacto-pyranoside. The method of the isolation of the wild-type hOGG1 was described previously [14], [21]. The protein concentration was measured according to the Bradford method [25]; the stock solution was stored at −20°C.

### Oligodeoxynucleotides

The sequences of ODNs used in this work are listed in Table 1. The ODNs were synthesized by standard phosphoramidite methods using an ASM-700 synthesizer (BIOSSET Ltd., Novosibirsk, Russia) in the Laboratory of Bionanotechnology of ICBFM from phosphoramidites purchased from Glen Research (Sterling, VA). Synthetic oligonucleotides were unloaded from the solid support with ammonium hydroxide according to the manufacturer protocols. Deprotected oligonucleotides were purified by HPLC. The purity of ODNs exceeded 98% as estimated by electrophoresis in 20% denaturing PAGE after staining with the Stains-All dye (Sigma-Aldrich). Concentrations of oligonucleotides were determined from their absorbances at 260 nm. The ODN duplexes were prepared by annealing of modified and complementary strands at the 1∶1 molar ratio in the reaction buffer (50 mM Tris-HCl (pH 7.5), 50 mM KCl, 1 mM EDTA, 1 mM dithiothreitol, 9% glycerol).

**Table 1:** Sequences of oligodeoxynucleotides used in this work.

Shorthand	Sequence*
oxoG	CTCTaPu**oxoG**CCTTCC/GGAAGGCCAGAG
G	CTCTaPu**G**CCTTCC/GGAAGGCCAGAG

*aPu, 2-aminopurine; oxoG, 7,8-dihydro-8-oxoguanosine.

### Stopped-flow Fluorescence Measurements

Stopped-flow measurements with fluorescence detection were carried out essentially as described previously [14], [20], [21]. A SX.18MV stopped-flow spectrometer (Applied Photophysics Ltd, UK) equipped with a 150 W Xe arc lamp and 2 mm path length optical cell was used. The dead time of the instrument is 1.4 ms. The fluorescence of aPu was excited at λ_ex_ = 310 nm and monitored at λ_em_>370 nm as transmitted by a Corion filter LG370. The concentration of substrates containing aPu in the reaction cell was 1 µM, and the concentration of hOGG1 protein was varied from 0.5 to 2.0 µM. Typically, each trace shown in the Figures is the average of four or more fluorescence traces recorded in individual experiments. All experiments were carried out in a buffer containing 50 mM Tris-HCl, pH 7.5, 50 mM KCl, 1 mM EDTA, 1 mM DDT and 9% glycerol (v/v) at different temperatures over the range of 10–30°C.

### Product Analysis

To analyze the products formed by hOGG1, the oxoG-substrate was 5′-^32^P-labeled using T4 polynucleotide kinase and [γ-^32^P] ATP. The reaction mixtures contained reaction buffer, 1.0 µM ^32^P-labeled oxoG-substrate and 2.0 µM enzyme. The reaction was initiated by adding the enzyme and allowed to proceed at different temperatures (10–30°C). Aliquots (2 µl) were withdrawn as required, mixed with 3 µl of gel-loading dye containing 7 M urea, and analyzed by 20% denaturing PAGE. The gels were exposed to Agfa CP-BU X-ray film (Agfa-Geavert), and the autoradiograms were scanned and quantified using Gel-Pro Analyzer, version 4.0.

### Data Processing

The processing of the fluorescence intensity traces was performed by numerical fitting of a solution of a system of ordinary differential equations corresponding to the reaction mechanism using DynaFit software (BioKin, Pullman, WA) [26] as described previously in [27]–[30].

The approach is based on the fluorescence intensity variation in the course of the reaction due to the sequential formation and further transformation of DNA-enzyme complex and its conformers. The software performs numerical integration of a system of ODE with subsequent non-linear least-squares regression analysis. The response factors of the intermediates (that are essentially the products of the molar extinction coefficients and the fluorescence quantum yields) are treated as fitting parameters in the data processing. In the evaluated mechanisms, except for the first bimolecular step, all other reactions are first order reactions. In the data processing, the kinetic information is obtained from the ‘temporal behavior’ of the fluorescence intensity, not from the ‘amplitudes’ of the specific signal contributions. Only the rate constants obtained in the non-linear fits were used to derive the thermochemical parameters. The ‘response factors’ for different conformers resulting from the fits were not used in the determination of the equilibrium constants, but rather provided additional information on the fluorescence intensity variations in different complexes and conformers.

Processing of individual kinetic curves does not unambiguously provide the kinetic parameters; therefore, global fits of sets of kinetic curves obtained at different concentrations of the reactants at each temperature was used. In the fits all relevant rate constants for the forward and reverse reactions, as well as the specific molar responses for all intermediate complexes were optimized.

Using the measured rate constants the equilibrium constants *K*_i_ (*k*_i_/*k*_–i_, *i* is the step number) were determined for G- and oxoG-substrate. The standard thermodynamic functions of the i-th equilibrium step were determined using the van’t Hoff equation, which represents the relation between the true thermodynamic equilibrium constant (*K_i_*) and the Standard Gibbs Energy, the Standard Enthalpy, and the Standard Entropy of the i-th reaction step [22]:
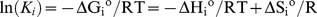
(1)


The typical dependence *ln*(*K*_i_) versus 1/T was linear, as expected for the relatively narrow temperature range of the study. The Gibbs free energies Δ*G°_i_* at 25°C were calculated from ln(*K*_i_) = −Δ*G°_i_*/RT. The validity of this approach was discussed elsewhere (e.g., Ref. [31]).

Analysis of the temperature dependence of the rate constant of a chemical reaction *k*_i_ permits determination of the standard enthalpy of activation (*Δ*H°^,‡^) and the standard entropy of activation (*Δ*S°^,‡^) based on the Transition State Theory (Eyring equation [22]). For unimolecular reactions, such as the catalytic step 5 in the reaction mechanism,
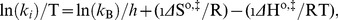
(2)where *k*_B_ and *h* are Boltzmann and Planck’s constants, respectively, *R* is the gas constant, *T* is absolute temperature in Kelvins, *k_i_* is the rate constant of the chemical step *i*.

### Molecular Modeling

The X-ray data obtained for complexes of hOGG1 with G and oxoG containing DNA ([17], PDB ID 1YQK and 1YQR, respectively), are taken as models of atomic structures of protein-DNA complexes (E•OG)_1_ and (E•OG)_3_ in Figure 3.

Inspection of these structures revealed, that structure 1YQK represents an example of the initial protein-DNA complex (E•OG)_1_, with bended DNA interacting with the protein and the G base placed in the exo-site. Structure 1YQR represents the specific protein-DNA complex (E•OG)_3_ with oxoG base everted from the double helix into the enzyme’s active site, with filled void in the double helix by Arg-154, Arg-204, Tyr-203 and Asn-149 and the enzyme forming a tight complex with the DNA-substrate in the catalytically active conformation. The contact areas ΔMS of the protein-DNA interface were calculated as the difference of the molecular surface (MS) confining the solvent-excluded volume [32] of isolated protein and the DNA and the protein-DNA complexes, ΔMS = MS (protein-DNA complex) – MS (protein) – MS (DNA), where the buried MS has negative sign. The MS surface was calculated by the SIMS method [33]. The free energy of the cavity formation in water in the process of desolvation has entropic nature and it is approximated by the linear equation ΔG_cav_ = γΔMS, where the parameter γ is in the range of 70–117 cal/mol/Å^2^ [32]. For the evaluation of the entropy loss, γ = 80 cal/mol/Å^2^ was accepted (which approximates the entropy of desolvation of non-polar species in water [34]).

## Results

### Interactions of hOGG1 with oxoG-substrate

**Figure 4:**
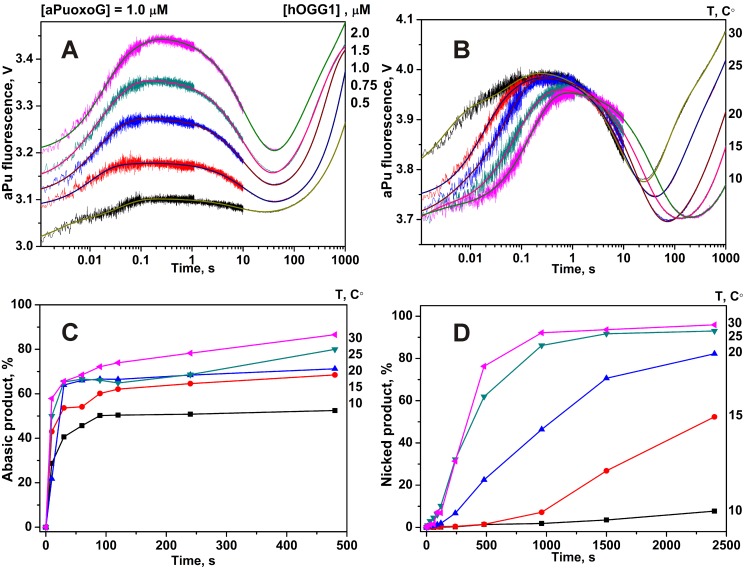
Stopped-flow fluorescence traces for interactions of hOGG1 with oxoG-substrate. Changes in aPu fluorescence intensity during interaction of hOGG1 with oxoG-substrate at different concentrations of enzyme at 25°C (A) and at different temperatures (B). Solid lines represent the fitted curves. The kinetics of the accumulation of the abasic (C) and nicked (D) products, formed in the N-glycosylase and AP-lyase reactions, respectively, as detected in the PAGE experiments. The concentrations of hOGG1 and DNA for (B), (C) and (D) panels were 2.0 µM and 1.0 µM, respectively.

**Figure 5:**
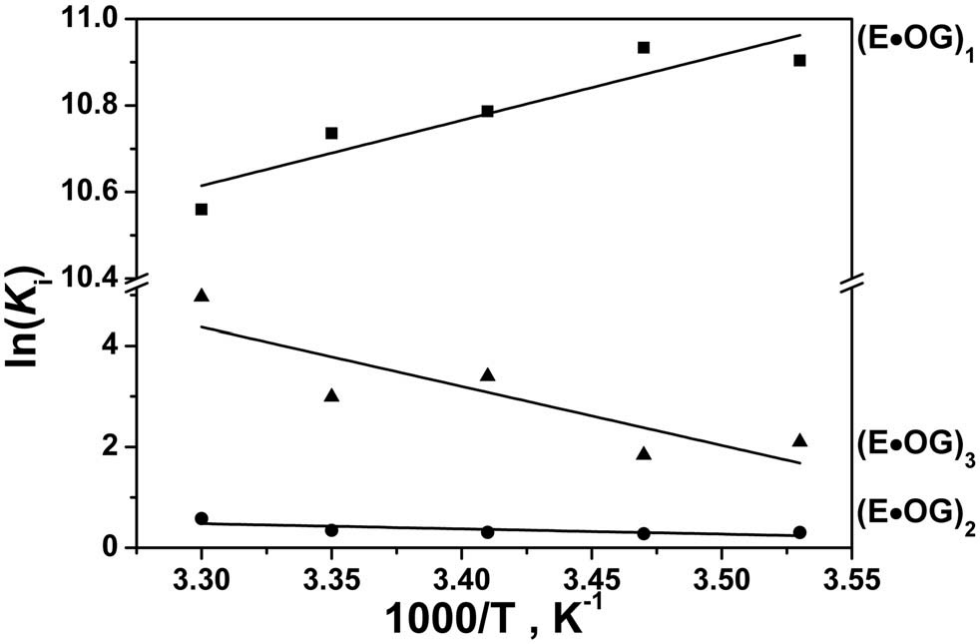
Van’t Hoff analysis of the temperature dependence of *K*i for oxoG-substrate.

The thermodynamics of the oxoG recognition by hOGG1 provided very important information for identification of the key steps in the DNA lesion search process. To quantitatively characterize the thermodynamics of the damage recognition process the changes in the fluorescence of an aPu label on 5′-side from oxoG base were used to reveal the conformational changes of DNA in the close proximity to the damaged base. The fluorescence traces measured for various concentrations of enzyme at each temperature (Fig. 4A and 4B) were fitted by the kinetic mechanism shown in Figure 3, as in the previous studies [14], [20], [21]. The rate constants obtained from the fits are summarized in Table 2. The thermodynamic parameters (Table 3) were then calculated using the van’t Hoff analysis (Fig. 5) as described in Materials and Methods.

**Table 2:** Pre-steady-state kinetic parameters for interaction of hOGG1 with oxoG-substrate^a^.

Rate constants	Temperature				
	10°C	15°C	20°C	25°C	30°C
*k*_1_, M^−1^s^−1^	(1.3±0.7)×10^7^	(2.2±1.0)×10^7^	(1.7±0.8)×10^7^	(1.9±1.0)×10^7^	(2.0±1.0)×10^7^
*k*_-1_, s^−1^	230±80	390±50	360±20	410±70	520±120
*k*_2_, s^−1^	8.3±3.8	15.6±4.2	26.6±6.0	53.5±20.3	72.4±31.4
*k*_-2_, s^−1^	6.1±0.2	11.8±0.2	19.6±1.7	37.9±11.1	40.5±12.5
*k*_3_, s^−1^	0.087±0.017	0.12±0.03	0.19±0.07	0.4±0.1	0.3±0.1
*k*_-3_, s^−1^	0.011±0.001	0.018±0.001	0.007±0.002	0.02±0.003	0.002±0.001
*k*_4_, s^−1^	0.005±0.001	0.007±0.001	0.014±0.002	0.026±0.003	0.041±0.007
*k*_5_, s^−1^	0.0006±0.0001	0.0012±0.0001	0.001±0.0001	0.001±0.001	0.0037±0.007

a The errors indicated are ±1 St. Dev.

**Table 3:** Thermodynamic parameters of hOGG1 interactions with oxoG-substrate^a^.

Stepnumber	Parameters			Processes	Possible structural detailsof the process in accordancewith [21]
	Δ*G*°_i298_,kcal/mol	Δ*H*°_i_,kcal/mol	Δ*S*°_i_,cal/(K*mol)		
1	−6.4	−2.8±0.7	11.2±2.4	nonspecific binding,DNA melting	C base pulling by Arg-154/Arg-204, pushing of Tyr-203 into DNA helix, oxoG flipping out into the exo-site, interaction of His-270 with everted base
2	−0.2	2.1±0.9	7.7±3.3	specific rearrangement ofthe enzyme-DNA complex, DNAkinking	oxoG flipping out in the active site, oxoG stacking with Phe-319, His-270 interaction with phosphate group of everted nucleotide, formation of hydrogen bonds between Arg-154, Arg-204, Asn-149 and C base
3	−1.8	23.2±7.8	85.4±26.6	final adjustment of the enzyme activesite to achieve catalyticcompetent state, DNAdesolvation	full insertion of Tyr-203 in DNA helix, oxoG interaction with Gly-42
Total	−8.4	22.5±9.4	104.3±32.3		
Transition stateof thecatalyticstep (4)	19.6	18.6±1.1	−3.5±3.9	N-glycosylasereaction	hydrolysis of covalent bond between atoms of ribose C1’ and oxoG N9
Transition stateof thecatalyticstep (5)	21.0	13.0±1.9	−27.0±6.7	AP-lyasereaction	3′-phosphodiester bond cleavage through β-elimination reaction

a The errors indicated are ±1 SD. The errors for the Gibbs Energies, δ(Δ*G*°_i298_) = RT(Δ*K*i/*K*i) ≤0.1 kcal/mol.

The effect of temperature on the rate of the chemical reactions was determined in the direct measurements of the rate of the products formation (abasic product in N-glycosylase reaction and DNA chain nick in AP-lyase reaction) using PAGE analysis of ^32^P-labeled oxoG-substrate. The results are shown in Figures 4C and 4D. The characteristic times of the abasic intermediate and nicked DNA product accumulation coincide with the characteristic times of aPu fluorescence increase in the 10–1000 s interval, indicating that the fluorescence changes in this time interval characterize the chemical steps of the enzymatic reaction.

### Interactions of hOGG1 with Non-specific DNA

**Figure 6:**
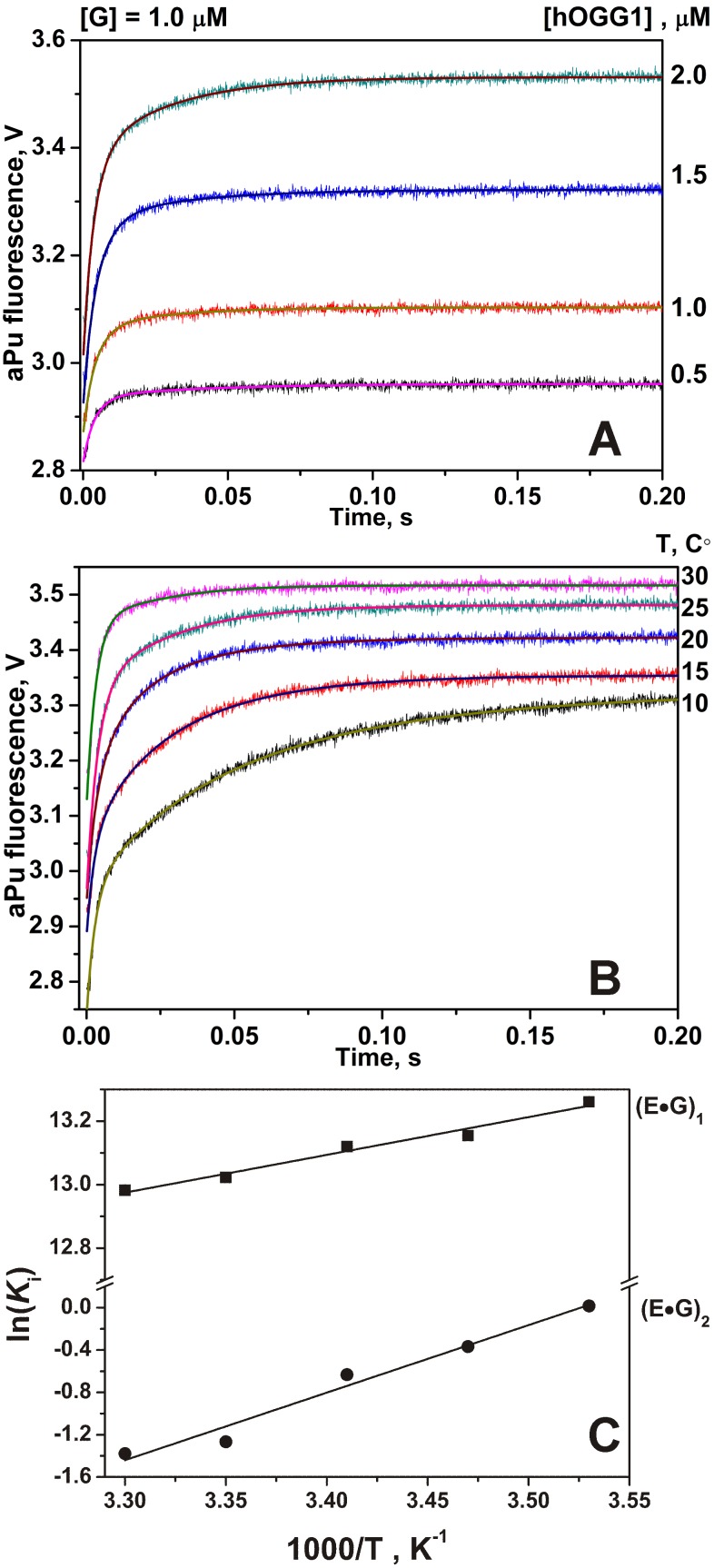
Stopped-flow fluorescence traces for interactions of hOGG1 with G-ligand. Changes in aPu fluorescence intensity during interaction of hOGG1 with G-ligand at different concentrations of enzyme at 25°C (A) and at different temperatures (B). Solid lines represent the fitted curves. The concentrations of hOGG1 and G-ligand were 2.0 µM and 1.0 µM, respectively. (C) Van’t Hoff analysis of the temperature dependence of *K*i for G-ligand.

The aPu fluorescence increases substantially during the process of hOGG1 binding of the non-specific G-ligand; the process is essentially completed in about 0.2 s (Fig. 6). The observed increase in the fluorescence intensity of aPu indicates local melting of the DNA chain interior of the non-specific enzyme-DNA complex. As it is known from the structural data obtained for covalently linked complex hOGG1 with a non-specific DNA, the enzyme is capable to flip out the undamaged G base not only in the exo-site [17] but also in the active site [19]. According to the kinetic data obtained for a set of hOGG1 mutant forms [21], the non-specific binding includes pulling of the opposite C base with Arg-154 and Arg-204, pushing by the Tyr-203 residue into the duplex between cytosine and the next base, the H-bond formation between the His-270 residue and the phosphate group of the damaged nucleotide, and stacking of the G base with the Phe-319 residue. All these factors lead to disruption of the stacking between aPu and G bases due to the flipping out of the latter and the DNA bending, which are expected to enhance aPu fluorescence. The aPu fluorescence traces obtained for G-ligand do not exhibit decreases in the fluorescence intensity as would be indicative of the void-filling processes. This means that the full insertion of the G base in the active site of hOGG1 as well as interaction of the opposite C with Arg-154, Arg-204, Asn-149 and Tyr-203 and its insertion into the DNA void, as suggested by the structural studies [19], is kinetically unfeasible.

**Figure 7:**
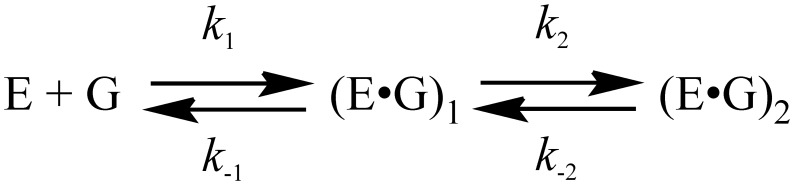
Binding of the non-damaged DNA by hOGG1.

The measured fluorescence traces at various concentrations of enzyme at each temperature (Fig. 6) were fitted by a kinetic mechanism containing two binding steps (Fig. 7). The forward and the reverse rate constants as well as the dissociation constant obtained at different temperatures are listed in Table 4. The thermodynamic parameters calculated as described in Materials and Methods are listed in Table 5.

**Table 4:** Pre-steady-state kinetic parameters for interaction of hOGG1 with G-ligand^a^.

Rate constants	Temperature				
	10°C	15°C	20°C	25°C	30°C
*k*_1_, M^−1^s^−1^	(5.6±1.2)×10^7^	(6.6±1.1)×10^7^	(5.9±1.3)×10^7^	(6.3±0.3)×10^7^	(7.4±1.4)×10^7^
*k*_-1_, s^−1^	97±23	128±21	119±19	140±14	170±17
*k*_2_, s^−1^	12.6±5.5	17.5±2.2	20.7±6.3	14.0±1.5	18.2±8.7
*k*_-2_, s^−1^	12.4±0.5	25.2±1.3	38.9±1.9	49.8±2.3	72.1±9.9

a The errors indicated are ±1 St. Dev.

**Table 5:** Thermodynamics parameters of hOGG1 interactions with G-ligand^a^.

Stepnumber	Parameters			Processes	Possible structural detailsof the process in accordancewith [21]
	Δ*G*°_i298_,kcal/mol	Δ*H*°_i_,kcal/mol	Δ*S*°_i_,cal/(K*mol)		
1	−7.7	−2.4±0.2	18.0±0.7	nonspecific binding, DNAmelting	C base pulling by Arg-154/Arg-204, partial pushing of Tyr-203 into DNA helix, G base flipping out into the exo-site, interaction of His-270 with everted base
2	0.8	−12.7±1.2	−44.7±4.1	the attempt to adjust thestructure ofactive site	DNA kinking, Tyr-203 “wedge” insertion
Total	−6.9	−15.1±1.4	−26.7±4.8		

a The errors indicated are ±1 SD. The errors for the Gibbs Energies, δ(Δ*G*°_i298_) = RT(Δ*K*i/*K*i) ≤0.1 kcal/mol.

## Discussion

Comparative analysis of the thermodynamic data presented in Tables 3 and 5 together with the structural information for free hOGG1 and various hOGG1/DNA complexes [9], [10], [15]–[19] provides important information on the energy consumption in the stages of the DNA binding, specific sites recognition and catalysis.

The process of G-ligand binding proceeds in two steps, which could belong to the formation of non-specific electrostatic and hydrophobic contacts with the sugar-phosphate backbone, DNA bending, Arg-154, Arg-204, Asn-149 and Tyr-203 amino acids insertion into the DNA and the G base flipping out into the exo- or active site of the hOGG1, as follows from the X-ray studies [17], [19].

The formation of the 1^st^ complex is favorable in terms of the Gibbs energy change, due to both negative enthalpy and positive entropy changes (Table 5). The second detectable binding step for G-ligand is characterized by the increase of aPu fluorescence and completed within ca. 0.2 s. As follows from Figure 6, no decrease in the aPu fluorescence was detected, indicating that the full insertion of mentioned above amino acids into the DNA void does not occur. The formation of a tighter 2^nd^ enzyme-ligand complex is characterized by the decrease of entropy compensated by a favorable change of enthalpy, indicating formation of new contacts, which stabilize this complex and make it more rigid (Table 5). Overall, binding of hOGG1 with G-ligand is thermodynamically favorable process with Δ*G°* = −6.9 kcal/mol.

Therefore, hOGG1 binding to G-ligand as monitored by direct fluorescence comprises melting of the duplex and eversion of the G base, with the G base left mainly outside the active center. This follows from the absence of kinetic evidence of full insertion of the enzyme amino acids into the DNA duplex, which is expected in the case when the G base is placed in the active site similar to that as for the oxoG base. Therefore, it is reasonable to conclude that in the case of true equilibrium and kinetically restricted state for the hOGG1/G-DNA complex the location of G base in the spatial structure could be different.

The fluorescence traces obtained for oxoG-substrate (Fig. 4A and 4B) show that at 0.2 s the aPu fluorescence starts to decrease, which characterizes the insertion of the amino acids. It means that the insertion of amino acids occurs immediately after the eversion of oxoG base from DNA-helix. Therefore, the second step is very important to discriminate G bases vs. oxoG bases. According to [21], specific interactions between Arg-154, Arg-204, Asn-149 and DNA chain occur during this step. Additionally, the oxoG is placed in the active site where it forms stacking structure with Phe-319.

For oxoG-substrate, the formation of the second (E•OG)_2_ and the third (E•OG)_3_ complexes in Figure 3 is characterized by the positive enthalpy and entropy changes (Table 3). A several processes during the protein/DNA complexation can lead to a significant unfavorable contribution to the enthalpy. The desolvation of polar groups at protein/DNA interface is expected to be energetically unfavorable [35]. Moreover, the protein bindings in the minor groove exhibit positive enthalpy and positive entropy changes that compensates the process endothermicity. Therefore, these processes are completely entropy driven [36]. The unfavorable enthalpy changes for the minor groove binding proteins result from the displacement of water molecules that are in the minor groove. Because these water molecules are highly ordered, their release into the bulk solution leads to a significant entropy growth. In the case of hOGG1, the aryl ring of Tyr-203 is wedged into the DNA duplex from the side of the minor groove [9]. Therefore these results are in accord with the conclusion that the enzyme interacts with the minor groove of oxoG-substrate [9].

Another potential source of unfavorable enthalpy Δ*H*° is the stress of the DNA structure induced by the ligand attachment. The comparison of the enthalpy and entropy changes for a wide range of DNA-binding proteins [35] shows that complexes in which the DNA is bent or severely distorted exhibit an unfavorable enthalpy changes compensated by favorable entropy changes. According to the structural data, in the catalytically active complex hOGG1 bends the DNA (ca. 70°) at the lesion site. Therefore, DNA bending can be also associated with these steps of oxoG-substrate binding by hOGG1.

The X-ray structures of the complexes of hOGG1 with G (1YQK) and oxoG (1YQR) containing DNA [17] might be considered as the atomic models for the hOGG1-DNA complexes corresponding to the initial complex (E•OG)_1_ and the catalytically competent complex (E•OG)_3_, respectively. Therefore, it was possible to estimate the change of the area of the protein-DNA interface during the formation of (E•OG)_3_ from (E•OG)_1_. The calculations show that the mutual adjustment of the enzyme and DNA in the complex (E•OG)_3_ is accompanied by the increase of the contact surface interface by 672 Å^2^ from 764 to 1436 Å^2^ in complexes (E•OG)_1_ and (E•OG)_3_, respectively. Burring of 672 Å^2^ of the molecular surface of the tight protein-DNA complex results in the entropy increase of about 180 cal mol^−1^ K^−1^ [34]. This entropy increase is partially compensated by the decrease of entropy due to the formation of the new tight protein-DNA contacts in the course of oxoG-substrate binding steps (Table 3).

The transition state for the catalytic step of the base excision (N-glycosylase reaction, step 4) as well as the 3′-phosphodiester bond cleavage (β-elimination reaction, step 5) has unfavorable enthalpy Δ*H°^,^* ^‡^ and favorable entropy ΔS°^,‡^ changes (Table 3). The results of the thermodynamic analysis of the catalytic steps obtained using aPu fluorescence data were qualitatively confirmed by the direct PAGE analysis of the products accumulation at different temperatures.

As it was recently demonstrated for another oxoG excision DNA glycosylase Fpg from *E. coli*, belonging to the Fpg/Nei family of DNA glycosylases, the determination of the thermodynamic parameters of the substrate binding and lesion removal steps provided important information about oxoG recognition process [27], [29], [30]. The pre-steady-state kinetic data were obtained using pyrrolocytosine fluorescence in DNA [27]. These data allowed determination of the Gibbs energy, enthalpy and entropy for the five rapidly proceeding steps of sequential damage recognition mechanism and to develop a detailed model of the Fpg enzymatic process. The first step had similar thermodynamic parameters both for undamaged G-ligand and oxoG-specific substrate, as it was obtained for hOGG1 catalysis in the present study. This step is highly exothermic. In this step the favorable enthalpy change is accompanied by an increase in entropy most probably due to the DNA melting in the point of contact and the conformational changes of the DNA binding site in the Fpg molecule with the emphasis on the contribution of the Phe-110 wedge movement. The next 2^nd^, 3^rd^ and 4^th^ steps are characterized by the negative or slightly positive ΔG° changes and reflect the formation of a kink in the DNA chain; eversion of oxoG base from the double helix into the enzyme’s active site; and filling the resulting void in the double helix by Arg-108 and Met-73. The 5^th^ reversible recognition step was characterized by a highly unfavorable enthalpy that was compensated by favorable entropic contribution. The large positive entropy of the protein binding originated from the dehydration of the DNA grooves and isomerization of the enzyme to form a tight complex with the DNA-substrate and to produce catalytically active conformation.

## Conclusions

Summarizing, in the present study a thermodynamic analysis of the reaction of human 8-oxoguanine DNA glycosylase with DNA duplexes containing undamaged and damaged nucleotides was performed. It was shown that the first binding step only weakly depends on the nature of the damaged nucleotide, supporting the hypothesis that the main contribution to the thermodynamics of this step is due to the non-specific interactions between the enzyme and the DNA backbone. The results of this study clearly demonstrate that the G base does not evert into the active site, however, do not exclude a possibility for the evertion of the G base in the exo-site suggested earlier by Verdine et al. [17]. The second step is crucial for the discrimination between oxoG and G bases. In the case of oxoG-substrate the second and the third steps have unfavorable enthalpy, which is compensated by favorable desolvation entropy. During these steps displacement of water molecules from the minor groove as well as DNA bending occurs. The last binding step also includes the final adjustment of the enzyme/substrate complex to the catalytically competent state. This step might be attributed to the full insertion of Tyr-203 in the DNA helix accompanied with the formation contacts between oxoG and Gly-42.

The thermodynamic analyses of the catalytic processes performed by functionally homologous but structurally different DNA-glycosylases, human OGG1 (this work) and *E. coli* Fpg [27], belonging to two distinct structural families, HhH-GPD and Fpg/Nei, respectively, demonstrate that they employ the common energetic features in the main steps of 8-oxoguanine lesion recognition. The first step of the DNA binding process is the formation of a non-specific complex, resulting in the double helix melting and insertion of wedging aminoacids (Tyr-203 for hOGG1 and Phe-110 for Fpg), characterized by favorable changes of both enthalpy and entropy. The following steps of lesion recognition are favorable in enthalpy but may be unfavorable in entropy due to the formation of structurally rigid complexes. The last step of the lesion recognition where enzyme-substrate complexes are rearranged to catalytically competent conformations is characterized by large endothermicity compensated by a significant increase of entropy originated from the dehydration of DNA grooves.
